# A systematic review of surgical intervention in the treatment of hamstring tendon ruptures: current evidence on the impact on patient outcomes

**DOI:** 10.1080/07853890.2022.2059560

**Published:** 2022-04-13

**Authors:** Aleksi Jokela, Antti Stenroos, Jussi Kosola, Xavier Valle, Lasse Lempainen

**Affiliations:** a Faculty of Medicine, University of Turku, Turku, Finland; b Department of Orthopedics and Traumatology, Helsinki University Central Hospital, Helsinki, Finland; c Department of Surgery, Kanta-Häme Central Hospital, Hämeenlinna, Finland; d Medical Department, FC Barcelona, Barcelona, Spain; e FinnOrthopaedics/Hospital Mehiläinen NEO, Turku, Finland

**Keywords:** Hamstring, muscle, tendon, biceps femoris, semitendinosus, semimembranosus, surgical repair

## Abstract

Hamstring injuries are among the most common muscle injuries. They have been reported in many different sports, such as running, soccer, track and field, rugby, and waterskiing. However, they are also present among the general population. Most hamstring injuries are mild strains, but also moderate and severe injuries occur. Hamstring injuries usually occur in rapid movements involving eccentric demands of the posterior thigh. Sprinting has been found to mainly affect the isolated proximal biceps femoris, whereas stretching-type injuries most often involve an isolated proximal injury of the semimembranosus muscle. The main cause of severe 2- or 3-tendon avulsion is a rapid forceful hip flexion with the ipsilateral knee extended. Most hamstring injuries are treated non-surgically with good results. However, there are also clear indications for surgical treatment, such as severe 2- or 3-tendon avulsions. In athletes, more aggressive recommendations concerning surgical treatment can be found. For a professional athlete, a proximal isolated tendon avulsion with clear retraction should be treated operatively regardless of the injured tendon. Surgical treatment has been found to have good results in severe injuries, especially if the avulsion injury is repaired in acute phase. In chronic hamstring injuries and recurring ruptures, the anatomical apposition of the retracted muscles is more difficult to be achieved. This review article analyses the outcomes of surgical treatment of hamstring ruptures. The present study confirms the previous knowledge that surgical treatment of hamstring tendon injuries causes good results with high satisfaction rates, both in complete and partial avulsions. Early surgical repair leads to better functional results with lower complication rates, especially in complete avulsions.

**KEY MESSAGEs**
- Surgical treatment of hamstring tendon ruptures leads to high satisfaction and return to sport rates.
- Both complete and partial hamstring tendon ruptures have better results after acute surgical repair, when compared to cases treated surgically later.
- Athletes with hamstring tendon ruptures should be treated more aggressively with operative methods.

## Introduction

Most of the hamstring injuries are seen in athletes during high-speed running or overstretching of the posterior thigh [1]. The most severe proximal hamstring injuries occur after rapid hip flexion with concurrent knee extension [2]. The hamstring complex consists of three muscles: biceps femoris (BF), semimembranosus (SM) and semitendinosus (ST) [3]. Anatomically, the origin of SM muscle is on the superolateral aspect of the ischial tuberosity underneath the proximal part of ST [4]. The ST originates from the inferomedial aspect of the ischial tuberosity and forms a common tendon together with the long head of BF [4]. However, anatomical variations occur, and ST can also have an isolated insertion area [5].

The degree of hamstring injury sustained can vary, ranging from a muscle strain to an avulsion of one or both tendinous insertions on the ischial tuberosity. Most hamstring injuries recover well with conservative treatment [6]. Complete rupture of the proximal hamstring complex has been defined as the tearing of all tendons with or without retraction [7]. Surgical intervention is recommended for complete avulsions, with or without retraction [8]. Treatment of incomplete proximal hamstring ruptures, involving one (most often SM) or two tendons (most often BF + ST), represents a challenging clinical problem to the surgeon [9,10].

The diagnosis of hamstring injury can be made in acute or chronic phase. Currently, there is no consensus about the definition of an acute injury, as the characterizations involve several different interpretations, such as <3, <4, <6, <8 or <12 weeks [11–15]. In addition to the fact that there are a wide variety of different diagnostic criteria for hamstring injuries, a consensus on the optimal treatment is lacking. Systematic reviews [8,16] have reported conflicting results when comparing outcomes of acute and chronic hamstring injuries.

To our knowledge, no previous systematic reviews on outcomes of surgical treatment of hamstring tendon ruptures, including studies with partial/incomplete and complete injuries located in all anatomical sites, have been published. Additionally, only little is known about the outcomes of surgical treatment in professional athletes [17]. The aim of this study was to analyse clinical and patient-reported outcomes of the surgical treatment of hamstring ruptures. Specifically, we sought to compare the outcomes of endoscopic vs. open treatment, acute vs. chronic repairs (<6 vs. ≥6 weeks), and partial/incomplete vs. complete repairs. We hypothesized that surgically treated complete proximal hamstring ruptures will benefit from the surgery, and early surgery leads to better results than surgery performed later.

## Methods

### Protocol and registration

This is a systematic review and meta-analysis, and the Preferred Reporting Items for Systematic Reviews and Meta-Analyses (PRISMA) statement was used when conducting and reporting this systematic review.

### Population

This study investigated exclusively skeletally mature patients aged 18 years or more with a surgically treated hamstring tendon (BF, ST and/or SM) rupture or avulsion confirmed intraoperatively and/or with ultrasound or magnetic resonance imaging. The subjects had to be followed-up using at least one clinical or patient-reported outcome measure (PROM).

### Search strategy

**Figure 1.**
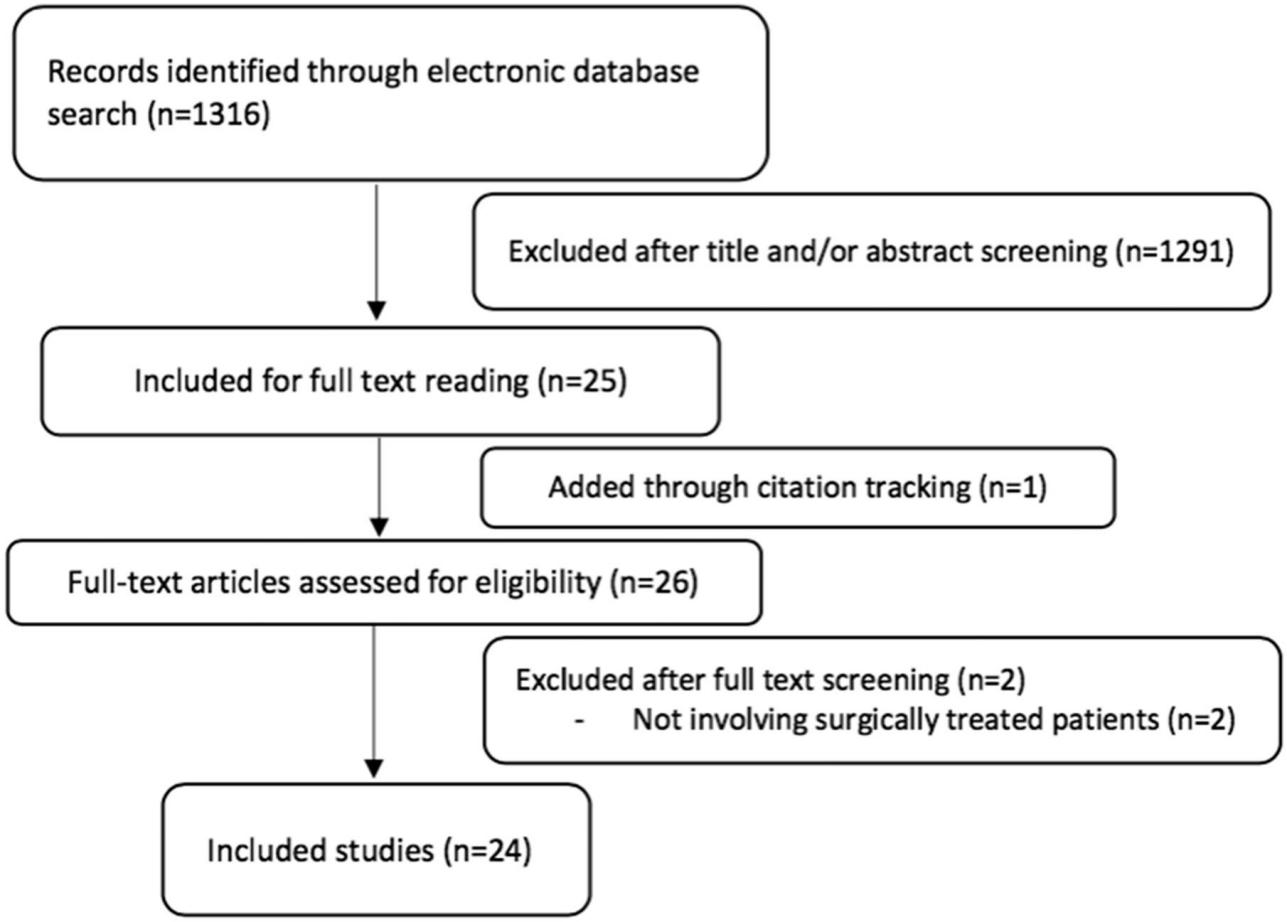
Selection process for included studies.

A systematic literature search was performed up to July 2021 in PubMed, CINAHL, Cochrane library, EMBASE, and Web of Science. The following keywords were used: “hamstring,” “avulsion,” “rupture,” “semitendinosus,” “semimembranosus,” “biceps femoris,” “femoral biceps,” “proximal,” “origin,” and “tendon.” Boolean operators “OR” and “AND” were used to combine synonyms and categories. The inclusion and exclusion criteria are presented in Table 1. Furthermore, we reviewed the reference lists of included publications and previously published review articles to identify any additional studies. Three reviewers (AS, JK, AJ) independently reviewed studies returned from the initial database search and resolved any disagreements by consensus; thereafter, two more authors (LL and XV) approved the inclusion (Figure 1).

**Table 1.** Inclusion and exclusion criteria for publications.

Criteria	Description
Inclusion	Published in a peer-reviewed journal in 2005 or later
	Studies that investigated surgically treated ruptures of BF, SM, ST or any combination confirmed intraoperatively and/or with US or MRI
	Studies that included patients aged 18 years or more
	Studies that had used at least one clinical or patient-reported outcome measure in follow-up
Exclusion	Studies that were not published in English
	Studies that included <15 patients
	Systematic and non-systematic reviews

### Data extraction and analysis

Each study was independently assessed by three reviewers (AJ, AS and JK). From each included publication, the following data were extracted to a customized worksheet: number of patients, mean age, method of treatment, follow-up time, return to sports, outcome measures and complications. These data were then checked independently by another author (LL). The data were then grouped into various categories that compared acute repair vs. chronic repair, partial/incomplete repair vs. complete repair, acute complete and repair vs. chronic complete repair. Complications were pooled into the following categories: rerupture, reoperation, infection/wound complications (including seroma and hypertrophic scarring), neurological complications (including transient sciatic nerve palsy, sciatica, numbness and paresthesias), peri-incisional numbness, deep venous thrombosis/pulmonary embolism and miscellaneous (including new-onset pain, haematoma, posterior thigh atrophy and complex regional pain syndrome).

### Data presentation

The data collected from the eligible studies were summarized in a textual format consistent with the information’s presentation within the original articles.

### Statistical methods

Point estimates were obtained by pooling estimated means and the corresponding standard deviations across studies. Weighted means and standard deviations were obtained from the studies considered. *p* Values for continuous variables were obtained by Mann–Whitney’s *U* tests and categorical variables by chi-square tests, and complications were compared through a test of difference in proportions. Analysis of variance (ANOVA) was used to analyse the differences in outcomes between acute and chronic repair groups. Significance was set at a *p* value of .05.

### Outcome measures

From the included studies, the outcomes were categorized and analysed independently. After reviewing the included studies, outcomes of surgical treatment were assessed. These outcomes were categorized into patient-reported outcomes, adverse events and clinical outcomes. Primary outcome measures were return to sport, satisfaction and Lower Extremity Functional Scale (LEFS). All outcome measures used in the included publication are listed in Table 2.

**Table 2.** All studies included for the review^a^.

Study	Age, years	Professional athletes (%)	Competitive athletes (%)	Recreational athletes (%)	Follow-up, months	Complete/ partial	Acute/ chronic	Outcome measures
Lempainen et al. [10]	33 (16–61)	13 (27.1)	15 (31.3)	19 (39.6)	36	0/48	6/42	Satisfaction, RTS
Sarimo et al. [15]	46 (18–71)	0	2 (4.9)	27 (65.9)	37	41/0	14/27	Satisfaction
McGregor et al. [28]	Men, acute: 31.4; men, chronic: 28.8; women, acute: 33; women, chronic: 34	NR	NR	NR	NR	22/7	12/17	RTS, VAS
Wood et al. [29]	40.2 (12.9–66.2)	NR	NR	NR	24	65/7	65/7	Strength, endurance, RTS
Birmingham et al. [32]	46 (19–65)	NR	NR	NR	43.3	23/0	9/14	Single-legged hop, strength, endurance
Cohen et al. [7]	47.7 (17–66)	NR	NR	NR	33	38/14	40/12	LEFS, C-LEFS, Marx, C-Marx, RTS, strength
Lefevre et al. [20]	39.3 (±11.4)	3 (8.8)	12 (35.3)	17 (50.0)	27.2	23/11	34/0	UCLA, Tegner, RTS, strength, satisfaction
Skaara et al. [11]	51	0	5 (16.1)	26 (83.9)	30	17/14	28/3	LEFS, PHIQ, strength, single-legged hop, RTS
Rust et al. [19]	Acute: 49.8 (25–74), chronic: 40.7 (14–62)	NR	NR	NR	45	72/0	51/21	SANE, SF-12, VAS, satisfaction
Barnett et al. [21]	42.5	NR	NR	NR	53.8	96/36	38/94	Strength, endurance, RTS, satisfaction
Subbu et al. [18]	29 (18–52)	0	112 (100)	0	12	112/0	78/24	RTS
Blakeney et al. [22]	50.5 (16–74)	NR	NR	NR	12	49/22	37/37	PHAT, SF-12, LEFS
Blakeney et al. [14]	50 (16–74)	NR	NR	NR	34	64/29	49/47	PHAT, VAS, RTS
Arner et al. [26]	47.3 (16–65)	1 (1.6)	4 (6.3)	59 (92.2)	78	0/64	36/28	LEFS, Marx, C-LEFS, C-Marx, strength, RTS, satisfaction
Best et al. [24]	NR	NR	NR	NR	28	NR	49/0	PHAT, C-LEFS, C-Marx
Bowman et al. [25]	51.1 (17–77)	NR	NR	NR	29	45/13	38/20	SANE, iHOT-12, KJOC Athletic Hip Score, satisfaction, VAS, Tegner, RTS
Pihl et al. [23]	51 (34–68)	NR	NR	31 (93.9)	49	29/4	NR	LEFS, PHIQ, VAS, satisfaction
Aldridge et al. [34]	42 (25–58)	NR	NR	NR	37.2	0/23	NR	VAS, RTS, satisfaction, strength, endurance
Wood et al. [31]	49.2 (21.5–74.4)	8 (5.1)	13 (8.3)	69 (44.2)	60	110/46	82/74	SHORE, PHAT
Kurowicki et al. [27]	46.2 (18–63)	0	0	20 (100)	NR	NR	NR	PROM, strength, VAS, UCLA, mHHS
Irger et al. [33]	49 (±13)	NR	NR	136 (51.7)	NR	208/55	213/50	NR
Kayani et al. [17]	26	34 (100)	0	0	24	34/0	34/0	RTS, satisfaction, ROM, strength, PROM, LEFS, Marx
Kayani et al. [13]	38.7	14 (34.1)	0	27 (65.9)	NR	0/41	0/41	RTS, satisfaction, ROM, strength, PROM, LEFS, Marx
Fletcher et al. [30]	52 (19–77)	1 (3.3)	3 (10.0)	18 (60.0)	44	17/13	2/29	iHOT-12, SANE, mHHS, HOS-ADL, RTS, satisfaction
Total	43.9	74 (12.2)	166 (27.3)	449 (49.6)	35.9	991/425	858/482	RTS (15/24), satisfaction (12/24), strength (9/24), LEFS (7/24), VAS (7/24), Marx (4/24), PHAT (4/24), endurance (4/24), C-LEFS (3/24), C-Marx (3/24), SANE (3/24), PROM (3/24), ROM (2/24), iHOT-12 (2/24), Tegner (2/24), PHIQ (2/24), SF-12 (2/24), UCLA (2/24), single-legged hop (2/24), mHHS (2/24), HOS-ADL (1/24), SHORE (1/24), KJOC Athletic Hip Score (1/24)

HOS-ADL: Hip Outcome Score, Activities of Daily Living; iHOT: International Hip Outcome Tool; KJOC: Kerlan-Jobe Orthopaedic Clinic; LEFS: Lower Extremity Functional Scale; mHHS: modified Harris Hip Score; NR: not reported; PHAT: Perth Hamstring Assessment Tool; PHIQ: Proximal Hamstring Injury Questionnaire; PROM: passive range of motion; ROM: range of motion; RTS: return to sport; SANE: Single Assessment Numeric Evaluation; SF-12: 12-Item Short Form Survey; SHORE: Sydney Hamstring Origin Rupture Evaluation; UCLA: University of California, Los Angeles; VAS: visual analogue scale.

^a^ Data are represented as a mean ± SD or mean (range).

## Results

### Publications

The literature search yielded 1316 reports after the exclusion of duplicates. After full-text review, 24 publications [7,10,13–15,17–33] were included (Figure 1). A thorough review of the bibliographies of the remaining studies was carried out and one additional publication [11] was identified through this method. Sixteen studies [7,11,14,15,18,19,21,22,25,26,28–33] included both acute and chronic ruptures. Three studies included only acute ruptures [23,24,34]. All but one study [17] consisted of proximal hamstring injuries. Twelve studies [7,11,14,20–23,25,28–31] included both complete and partial ruptures. Five studies included only complete [15,17,19,32,33] and three only partial [10,13,26,34]. These data were not available in the remaining studies.

### Demographic data

The study population comprised 1602 patients with hamstring injuries. The mean age of the patients in the included studies ranged from 26 to 51 years, and the weighted average age was 44.8 (range, 16–77) years, at the time of injury. The mean follow-up ranged from 12 to 78 months (Table 2).

### Open vs. endoscopic

Majority of patient were treated with open surgery (1548, 96%) and 62 (4%) with endoscopic approach. Most patients were treated with primary repair. However, a total of 24 chronic complete avulsions were treated with repair augmented with graft.

### Outcome measures

Return to sports was used in 62.5% of the included studies, patient satisfaction in 50.0%, and strength in 37.5%. Of the two validated clinical assessment tools specified to hamstring repair, Perth Hamstring Assessment Tool (PHAT) was used in 16.7% of the studies and Sydney Hamstring Origin Rupture Evaluation (SHORE) in 4.2%. LEFS and visual analogue scale (VAS) were present in 29.2%, whereas Marx, PHAT and endurance were involved in 12.5% of the publications. All outcome measures used in the included publication are listed in Table 2.

### Satisfaction, return to sport and complications

After repair, 89% of patients were satisfied with their outcome. In total, 80% (range 62.5–100%) of the patients returned to sport on same level. Total rate of complications was 15.69% (Table 3). The complication rate varied between studies from 0 to 50% due to heterogenic definition of complication. Miscellaneous complications were most common (32.9%), followed by neurologic complications (24.9%), peri-incisional numbness (16.9%) and infection/wound complications (14.1%). Complications for operatively treated acute and chronic, and partial and complete hamstring avulsions are presented in Tables 4 and 5.

**Table 3.** Complications for operatively treated hamstring avulsions.

	Incidence, %	No.
Rerupture	0.69	11
Reoperation	0.50	8
Infection/wound complications	2.21	35
Neurologic complications	3.91	62
Peri-incisional numbness	2.65	42
DVT/PE	0.57	9
Miscellaneous	5.17	82
Total	15.69	249

**Table 4.** Complications for operatively treated acute and chronic hamstring avulsions.

	Acute, %	No.	Chronic, %	No.
Rerupture			1.06	2
Reoperation	0.33	1		
Infection/wound complications	1.99	6	1.06	2
Neurologic complications	1.66	5	9.04	17
Peri-incisional numbness	5.32	16	2.13	4
DVT/PE	0.66	2	1.06	2
Miscellaneous	3.99	12	10.64	20
Total	13.95	42	25.00	47

**Table 5.** Complications for operatively treated partial and complete hamstring avulsions.

	Partial, %	No.	Complete, %	No.
Rerupture	0.45	1	1.02	4
Reoperation	1.81	4	0.77	3
Infection/wound complications	2.71	6	3.57	14
Neurologic complications	3.17	7	8.67	34
Peri-incisional numbness	2.71	6	3.57	14
DVT/PE	0.90	2	0.77	3
Miscellaneous	9.95	22	6.12	24
Total	21.72	48	24.49	96

### Acute vs. chronic

Acute avulsions were arbitrarily defined as patients who were operatively treated within 6 weeks after the injury, while chronic avulsions underwent surgery after 6 weeks and analysis was done from studies where comparison was possible [7,13,14,17–21,23,24,31,35] (815 patients, 502 acute and 313 chronic). The mean time from injury to intervention for the acute and chronic groups was 2.8 and 46 weeks, respectively. After repair, 95% of patients acute patients were satisfied with their outcome, which was significantly greater (*p* < .001) compared to chronic group (77%). Similar finding was noted in return to sports in same level (92% vs. 85%, *p* < .001). Analysis of variance suggested the effect of time to surgery on return to sport was significant. Weighed mean return to sports was 4.5 months in acute group and 5.6 months in the chronic group (*p* < .001) No statistically significant differences were found in LEFS (74 vs. 72) at 1 year. Compared with the chronic group, acutely treated patients reported significantly better results (*p* < .001) in strength testing comparing to the contralateral leg.

### Partial/incomplete vs. complete

A partial/incomplete avulsion was defined as detachment of 1 or 2 (BF + ST) hamstring tendons (completely or partially), with the remainder of the musculotendinous complex still intact. Partial avulsion was registered in 270 patients and included in analysis weighted mean age 42.5 years, complete avulsion in 293 patients with weighted mean age 41.7 years [10,15,17–19,21,26,30–32,34]. After repair, 92% of patients with complete ruptures were satisfied with their outcome, and 87% with partial ruptures. Complete avulsions had lower pain scores (1.87 vs. 3.76), (*p* < .001). Return to sports in same level was reported on 78% with partial avulsions and 81% complete avulsions. Patients who underwent partial repair had higher strength testing (89% vs. 88%, *p*>.05).

### Partial acute vs. partial chronic

Separate analysis for partial chronic and partial acute avulsion was attempted but no reliable comparisons could not be made from the available data due to the limited amount of reported cases.

## Discussion

This is the largest systematic review researching the current evidence of patient outcomes after surgical treatment of hamstring tendon ruptures. The main finding is that early surgical intervention leads to high satisfaction and good functional outcomes. In addition, acute repair leads to better results and lower complication rates, when compared to chronic injuries. Furthermore, we noted that some clinically important issues, such as the amount of tendon retraction and individual tendon concept, have not been thoroughly addressed in the current literature.

Two previously published systematic reviews have reported better outcomes after acute repair of hamstring rupture when compared to delayed, chronic repair [8,16]. On the other hand, van der Made et al. [12] reported minimal to no differences in outcome of acute and delayed repairs with similar results in satisfaction, pain, functional scale scores and strength/flexibility. Belk et al. [36] found that the early repair group had the quickest time to return to sports and the highest rate of return to sports, but statistical significance was not reached in neither of these outcomes. Coughlin et al. [37] concluded that no major differences were found in return to sports between acute and chronic groups, discussing that the definition of chronicity varied between studies, which may have influenced the results. In this study, we found acute repair resulting statistically significantly better outcomes in satisfaction (*p* < .001), return to sports (*p* < .001) and strength (*p* < .001). This systematic review included 858 acute and 482 chronic repairs, as acute injuries were more commonly complete avulsions and chronic repair was more often performed in partial/incomplete injuries. As mentioned before, complications were more common after chronic than acute repair. There are several possible reasons to better outcomes in acute repair compared to chronic cases. In a technical point of view, acute repairs tend to be less demanding than chronic cases as they involve lesser amount of scar tissue at the area of injury and the anatomical structures are more easily distinguished. The scar tissue can also cause chronic symptoms due to the proximity of the sciatic nerve and pressure effect, which can cause local pain and tenderness. In addition, a great tendon retraction can also be found in chronic cases, which may be among the factors causing better results of acute repairs. However, some patients clearly benefit from the surgical treatment of the chronic hamstring tendon rupture, if they remain symptomatic even after a long period of rehabilitation [10].

In studies included in this systematic review, most commonly used outcome measures were return to sports (62.5%), patient satisfaction (50.0%) and strength (37.5%). As there is currently no consensus on the best outcome measures in the evaluation of patient outcomes after surgical repair of hamstring injury [38], there is a wide spectrum of different, mostly non-specific and nonvalidated, outcome measures used in studies investigating results of hamstring repair. Patient-reported outcome measures and clinical outcome measures (COMs) are important tools for evaluation of the results of surgical treatment, and the heterogeneity of outcome measures leads to difficulties in comparison of different studies and therefore in generalization of the results. To date, only two clinical assessment tools have been validated specifically for outcomes of hamstring repair, PHAT [22] and SHORE [39]. These outcome measures are recently developed and validated specifically for evaluation of hamstring injuries, and there are only limited amount of studies that have used these tools. In this systematic review, only 4/24 (16.7%) studies had used PHAT and only one study (4.2%) involved SHORE. The other validated and non-specific outcome measures, that are frequently used in assessment of outcomes after hamstring repair, include LEFS, 12-Item Short Form Health Survey (SF-12), VAS for pain, Single Assessment Numeric Evaluation (SANE) and Tegner Activity Scale (TAS) [38]. These outcome measures are valid and multifunctional that can be used in a wide range of purposes, but the usability and usefulness specifically in hamstring injuries is somewhat questionable. Therefore, we highly recommend the use of validated hamstring-specific outcome measures after hamstring repair, in addition to return to sports and isokinetic strength testing [38].

A systematic review authored by Bodendorfer et al. [8] compared outcomes between operative and nonoperative treatment, and they found operative patients having significantly higher scores in most categories, such as satisfaction, strength, single-legged hop test and LEFS (*p* < .001). In addition, Harris et al. [16] performed a systematic review concluding that compared to nonoperative patients, surgically treated patients reported significantly (*p* < .050) better strength testing, endurance levels and return to sports. However, the nonoperative groups were relatively small in both of these studies, making it difficult to reliably compare the outcomes between surgical and conservative treatment groups. In this study, 89% of the patients were satisfied with the outcome after operative treatment. In total, 80% of the patients returned to the same level of sports.

Complications related to surgical treatment of proximal hamstring avulsions have been noted to be relatively high in prior systematic reviews, despite the successful outcomes. Bodendorfer et al. [8] reported that complications occurred in 23.17% of cases. The research group found complete repairs having significantly higher complication rates than individual one- or two-tendon repairs (*p*=.001), and chronic repairs being associated with higher rates of neurologic complications (*p*=.051). In addition, both partial and complete repair groups reported postoperative sitting pain (7.04% and 9.38%). Harris et al. [16] concluded that acute surgical repair of proximal hamstring ruptures has low risk of complications and rerupture. van der Made et al. [12] reported complication rate of 28.68% after surgical repair of proximal hamstring avulsions. However, risk of major complications (deep vein thrombosis, wound infection, postoperative haematoma and symptoms of stiffness or numbness/tingling) was low. In this study, total incidence of complications was 15.69%, miscellaneous and neurologic complications being the most common ones and complication rate being higher in chronic repairs, which is consistent to previous studies. Chronic repairs involved complication rate of 25.00%, whereas 13.95% of acute repairs reported complications. Neurologic complications were also more common in chronic group (9.04% vs. 1.66%), miscellaneous complications presenting similar findings (chronic: 10.64% vs. acute: 3.99%). Compared to acute ruptures, chronic injuries often involve greater amount of scar tissue, which can also adhere to the sciatic nerve causing neurological symptoms. Therefore, chronic repairs tend to be more demanding leading to poorer outcomes and higher risk of complications. Complication rates were relatively similar in complete and partial avulsions, as in complete group 24.49% reported complications and 21.72% of the partial patients had complications. Interestingly, operatively treated chronic complete avulsions had a complication rate of 31.58%, whereas acute complete group reported 8.93% of patients having complications. However, the number of patients in both groups was small, so the statistical and clinical relevance of this finding remains open. The heterogeneity in complications’ reporting criteria and lack of clear classification between minor and major complications may explain high complication rates and great variation between different studies.

The most severe hamstring injuries often have similar injury mechanisms. Powerful overstretching of the hamstrings caused by a rapid hyperflexion of the hip with the ipsilateral knee in extension often cause proximal hamstring avulsion injuries [18,40]. Often in such injuries total energy is high, and there is a lack of control in the movement, which leads to a sudden hyperflexion of the hip. This is the main cause of severe 2- and 3-tendon avulsions, whereas proximal hamstring tendon avulsions following unconventional injury mechanisms are not so well documented [41]. In general, both the knee and hip joints are stabilized by eccentric contractions of the hamstring muscles. During a forced flexion of the hip with the knee extended, hamstring muscles are passively stretched and contracted eccentrically. During eccentric contractions, injuries to the muscle and proximal complex are more common [42]. This kind of injury mechanism should make a clinician to suspect a severe proximal hamstring avulsion injury, which seems to benefit from early diagnostics and surgical repair [18,41].

In this systematic review, 74 patients (12.2%) were professional athletes, 166 (27.3%) competitive athletes and 449 (49.6%) recreational athletes. The mean age of all studies was 43.9 years. A study by Kayani et al. [17] was the only one reporting results of hamstring repair in only professional athletes (*n* = 34), mean age being 26 years. They investigated the results of surgical treatment in acute injuries to the distal part of the BF, concluding that surgery led to high satisfaction, increased muscle strength, improved functional outcome scores and high return to preinjury level of sporting activity with low risk of recurrence [17]. A study by Subbu et al. [35] involved only competitive level athletes (*n* = 112, mean age 29 years) and they found that early operative intervention of complete proximal hamstring avulsion was associated with good clinical outcomes and a quicker return to sports. On the other hand, in a group in which the diagnosis and surgical treatment were delayed, prolonged morbidity and complications were more common [35]. Another study by Kayani et al. [13] investigated the outcomes after surgical repair of chronic partial proximal hamstring avulsions in professional (*n* = 14) and recreational athletes (*n* = 27), mean age being 38.7. They found that patients had high satisfaction and improved isometric hamstring muscle strength, range of motion, and functional outcome scores when compared to preoperative values, the length of follow-up being 2 years [13]. In conclusion, surgical repair of hamstring tendon injuries seems to lead to good outcomes, especially in high level athletes and active patients. However, studies involving professional or competitive level athletes are scarce and involve a low number of patients. Therefore, further research focussing on the results surgical repair of hamstring injuries in high-level athletes is needed.

As we mentioned earlier, no consensus has been reached to define an acute injury as there are currently several ways to categorize acute and chronic hamstring injuries. Based on our clinical experience, acute injury should be defined as being diagnosed within three weeks after injury, especially in complete proximal avulsions. If the proximal tendon end is retracted remarkably, the surgery is often even more important to be performed in acute phase. In clinical work, from 4 weeks to 8 weeks delay is most often already very chronic injury, especially if the tendon is clearly retracted. Therefore, we want to emphasize the importance of early diagnostics and decision-making in terms of treatment, as chronic repairs tend to have poorer outcome in proximal hamstring avulsions. Early operative treatment is important especially in high-level athletes with clear tendon retraction as the physical disability may cause more harm to patients with high demands. On the other hand, partial hamstring ruptures are more often treated first conservatively and operative treatment is often chosen after the failed rehabilitation. The tendon retraction is not as often as remarkable in partial injuries than in complete avulsions. Therefore, the clinical picture may be better for a longer period and the definition of injury chronicity should probably be slightly different in partial injuries than complete ruptures. However, the terms acuity and chronicity should be discussed more on the field of orthopaedics, as currently there is a wide variety of different categorizations on this matter. The consensus in definitions would probably lead to better results as treatment guidelines would also be more consistent.

Bodendorfer et al. [8] reported partial avulsion group demonstrating significantly better scores on strength and endurance testing (*p* < .001), whereas the complete avulsion group showed greater patient satisfaction (*p* < .001) and lower reported pain levels (*p* < .001). It is notable that partial injuries are more often chronic as they have traditionally been treated nonoperatively, with the exception of failure of 6-month conservative treatment, after of which operative treatment is often recommended [9,43]. Therefore, the chronicity of partial injuries may cause confounding bias in the analyses of these injuries. Belk et al. [36] concluded in a systematic review that patients with partial and complete hamstring tears can be expected to return to sports at a similar rate after operative repair (partial 96.8% and complete 93.0%, *p*=.18), which was similar finding with the systematic review authored by Coughlin et al. [37]. In this study, most of the patients were satisfied in both groups (complete 92% vs. partial 87%). Complete avulsion repair led to lower pain scores (*p* < .001), whereas statistically significant difference between groups was not reached in return to sports and strength testing. However, partial injuries are more often treated conservatively and they tend to cause chronic problems, eventually leading to need of surgical intervention. We recommend to assess carefully the possible need of surgery, not only in complete avulsions, but also in partial hamstring tendon ruptures, especially in athletes and active patients.

The amount of tendon retraction is an important factor when deciding the treatment method [29]. Based on our experience and the previous literature, severe two- and three-tendon avulsions with clear retractions are recommended to be treated operatively [16,44]. The threshold value for surgical treatment is often recommended to be minimum of 2 cm of tendon distal retraction [16,44]. Therefore, most of the acutely treated cases have 2 cm or more tendon retraction. Supposedly, the tendon retraction is less than 2 cm in most of the chronic cases as the surgery is performed after unsuccessful conservative treatment. The tendon can also retract during the rehabilitation process and the retraction value can meet the criteria of surgical treatment only after a long period of rehabilitation [29]. However, no studies further analysed the correlation between the amount of tendon retraction and outcomes after operative treatment. Although the tendon retraction is often lesser in partial ruptures, these injuries can cause difficult and persistent symptoms [43]. This may happen due to the scar tissue and adhesions, which are often present after recurrent injuries [45]. It is recommended to carefully choose the treatment methods based on individual information and patient’s demands [46]. For example, some patients with high physical demands, such as athletes, may benefit from the surgery even with lesser tendon retraction than 2 cm [43,46]. Sometimes proximal non-retracted partial avulsions remain symptomatic, despite the optimal conservative treatment and lack of retraction [29,43]. In these cases, the MRI may show fluid between ischial tuberosity and the injured tendon attachment [47]. This may be a sign of incomplete healing process and the surgical treatment can be indicated [43]. When it comes to partial/incomplete hamstring tendon ruptures, it is reasonable to highlight the individuality of each tendon [48]. Although each tendon has their individual functional characteristics [49], there are no clear evidence-based treatment guidelines concerning isolated BF, ST or SM ruptures.

One major limitation of this review is that most of the included studies had low methodological quality involving limited period of follow-up and lacking randomization or blinding. Additionally, there were wide variety of outcome measures, and totally 23 different outcome measures were used in included studies. This makes it difficult to generalize and aggregate the results from different studies. The categorization of complications was also heterogeneous as specification of the severity and nature of complication was lacking in many studies. The included patient population varied from professional athletes to regular patients, involving both males and females, age ranging from 14 to 77 (mean age 43.9). Therefore, the patient population was very heterogeneous and only few studies reported properly the chronicity, severity, activity level and other important details. The patient population involved 74 professional athletes, 166 competitive level athletes and 449 recreational athletes, as the level of sports was not reported for the rest, and the most, of the patients. The subgroup analysis for the different groups categorized into different activity levels would be essential in future studies to develop the management of hamstring injuries, especially in athletes. For now, the heterogeneity of patients can cause bias in outcomes making the interpretation of the results challenging. Additionally, some clinically important factors such as the amount of tendon retraction and individual tendon concept, have not been thoroughly addressed in the previous literature. Investigating these two essential entities would be crucial in future studies, in order to develop the clinical management of different hamstring injuries.

This study has also remarkable strengths, including a large sample size with 1602 operatively treated patients with hamstring tendon injury. A large patient population makes the generalizability of the results more reliable. In terms of patient population, this is the largest systematic review investigating the outcomes of surgical repair of hamstring tendon injuries.

In conclusion, most of the patients are satisfied with the result and return to sports after surgical treatment of hamstring tendon injuries, both in complete and partial avulsions. Compared to chronic repair, early surgical intervention leads to better results with higher satisfaction and better functional outcomes. In addition, complications are significantly more common after chronic repair. After early surgical treatment of hamstring tendon injuries, a good functional outcome can be expected. Additionally, complete hamstring tendon repairs have better results when compared to partial repairs.

## Authors contributions

LL was responsible for the conception. All authors were responsible for the design of the study. AJ, AS and JK were responsible for the acquisition of the literature for the manuscript. AS was responsible for the statistical analysis. All authors were involved in interpretation of the data. AJ and AS wrote the original draft of the manuscript. JK, XV and LL revised the paper critically for intellectual content. XV and LL supervised the paper. All authors have read and approved the final version to be published. All authors agreed to be accountable for all aspects of the work.

## Data Availability

Data sharing is not applicable to this article as no new data were created or analysed in this study.
